# Oncocytic Schneiderian papilloma-associated adenocarcinoma and KRAS mutation: A case report

**DOI:** 10.1097/MD.0000000000011025

**Published:** 2018-06-18

**Authors:** Lichuan Zhang, Chunhua Hu, Xiaodan Zheng, Dawei Wu, Haili Sun, Wei Yu, Ying Wu, Dong Chen, Qianwen Lv, Ping Zhang, Xiping Li, Honggang Liu, Yongxiang Wei

**Affiliations:** a Department of Otolaryngology–Head and Neck Surgery, Beijing An Zhen Hospital, Capital Medical University; b Department of Pathology, Beijing Friendship Hospital; c Department of Pathology, Beijing An Zhen Hospital; d Department of Pathology, Beijing Tong Ren Hospital, Capital Medical University, Beijing, PR China.

**Keywords:** adenocarcinoma, KRAS, oncocytic, schneiderian papilloma, sinonasal

## Abstract

**Rationale:**

Oncocytic Schneiderian papillomas (OSP) are an uncommon type of sinonasal papillomas that arise from the Schneiderian epithelium, accounting for only 6% of all sinonasal papillomas. Malignancies arising in OSP are rare and are almost always described as in situ or invasive squamous cell carcinoma, although mucoepidermoid, small cell carcinoma and sinonasal undifferentiated carcinoma have also been reported. To our knowledge, only 18 such instances have been reported in the medical literature.

**Patient concerns:**

Here, we report the case of an 81-year-old man presenting with a left sinonasal neoplasm, who had undergone 4 operations. The first postoperative pathology revealed a benign nasal polyp. The following several postoperative pathology revealed a novel, human papillomavirus-negative adenocarcinoma with increasing malignant features with each recurrence arising in an OSP. In addition, the most recent recurrences were associated with metastasis of cervical lymph nodes. And after the operation, the patient refused adjuvant radiotherapy. On 6-month follow-up after the last operation, the patient developed an in situ tumor recurrence 1 month after the fourth operation and refused to undergo surgery again.

**Diagnosis:**

Immunohistochemistry for Ki67, CK7, CK5/6, P53, and P63 showed a progression of malignancy. HPV assay presented the 21 most prevalent HPV types were negative. In addition, KRAS gene exon 2 G12C presented mutation in the OSP-associated adenocarcinoma.

**Interventions:**

During the whole course of the patient's disease, we performed four nasal endoscopic operations. And after the last operation, the patient refused adjuvant radiotherapy and KRAS-targeted therapy.

**Outcomes:**

We are the first to describe adenocarcinoma arising in an OSP. To our surprise, from the first benign neoplasm to the second OSP-associated adenocarcinoma, it went through a long period of 10 years. However, after the adenocarcinogenesis, the differentiation of tumor became worse with the shorter interval of each recurrence.

**Lessons:**

Therefore, for elderly patients with unilateral nasal polyps, long-term follow-up is necessary. Once OSP turns into malignant, radical resection should be performed as much as possible to reduce the irritability of tumors.

## 1 Introduction

Schneiderian papillomas are uncommon benign neoplasms derived from the Schneiderian epithelium, which is an ectodermally derived ciliated respiratory epithelium that covers the surface of the nasal cavity and paranasal sinuses. These tumors account for approximately 0.4% to 4.7% of sinonasal tumors.^[1]^

The World Health Organization (WHO) defines 3 types of Schneiderian papillomas: exophytic papillomas (EP), inverted papillomas (IP), and oncocytic Schneiderian papillomas (OSP). The morbidities of the 3 types of papillomas are quite different.^[2,3]^ Hyams^[4]^ reported the proportion of these tumors as EP 49.5% and IP 47.3%, and OSP 3.2%. OSPs are the rarest of the 3 subtypes.^[2]^ Human papillomavirus (HPV) can be found in IP and EP, but not in OSP.^[5,6]^ Therefore, OSP is considered to have a different pathogenesis, although the initial clinical manifestations are similar. All 3 types can progress to carcinoma, especially IP and OSP. The most common type of carcinogenesis in Schneiderian papillomas is squamous cell carcinoma,^[7]^ and there are also reports of mucoepidermoid carcinoma, small cell carcinoma, and sinonasal undifferentiated carcinoma.^[3,7,8]^ Some reports have estimated a prevalence of carcinoma development in as high as 10% to 15% of IP cases.^[9]^ OSP also has a risk for malignancy, but malignancies arising in OSPs are rare, with invasive squamous cell carcinoma being the most frequently reported tumor, ^[4,10–12]^ Given the rarity of these cases, the true rate of malignancy is hard to assess.^[10]^ To the best of our knowledge, only 18 such instances (Table 1) have been reported in the medical literature.^[13–15]^

**Table 1:**
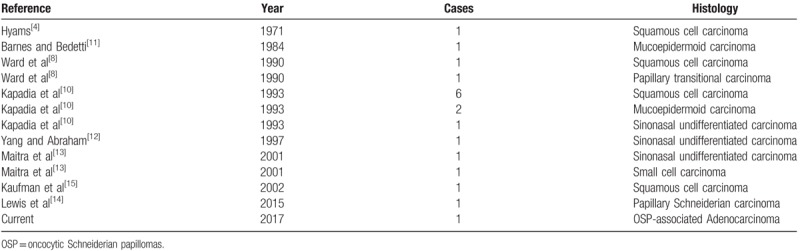
Malignancies Arising in Oncocytic Schneiderian Papillomas.

To date, there is no report on adenocarcinoma arising in the papillomas of the nasal cavity and paranasal sinus. Also, there are few reports on the biological behavior of OSP-related tumors after malignant transformation, such as in recurrence, lymph node metastasis, and distant metastasis.^[14]^ Only 1 case described cervical lymph node metastasis after carcinoma arising in IP.^[14]^ To our knowledge, metastasis following malignant transformation arising in OSP and the progress of the disease have not yet been reported.

Pathological diagnosis is the criterion standard for diagnosis and plays a vital role in the treatment and prognosis of Schneiderian papillomas. For patients with multiple surgeries and recurrences, dynamic observation of the pathological changes is helpful to guide treatment decisions.

Here, we report a longitudinal study on a patient with OSP-related adenocarcinoma who required multiple surgeries and allowed for analysis of the recurrent pathology. Combining clinical phenotypes with pathological characteristics and genotypes helped provide guidance for diagnosis and treatment for this case.

## 2 Materials and methods

### 2.1 Ethics review and patient consent

This case report dealt only with the patient's clinical records. Ethics committee approval was not thought to be necessary because the entire clinical course of the case was within standard medical care. Informed consent on diagnosis and treatment was given by the patient.

### 2.2 Patient

A 69-year-old Chinese man presented in 2005 with left nasal obstruction and occasional epistaxis after a cold to the Otolaryngology clinic at the Peking University People's Hospital of China, a tertiary care academic center in Beijing, China. He denied rhinorrhea, headaches, and ophthalmic or neurological symptoms. His medical history was remarkable for hypertension and coronary heart disease. Endoscopic examination revealed a large left sinonasal mass, and imaging confirmed the absence of bony erosion into the orbit or skull base. We performed endoscopic extirpation of the left nasal polyp under local anesthesia. Postoperative pathology revealed a benign nasal polyp.

**Figure 1:**
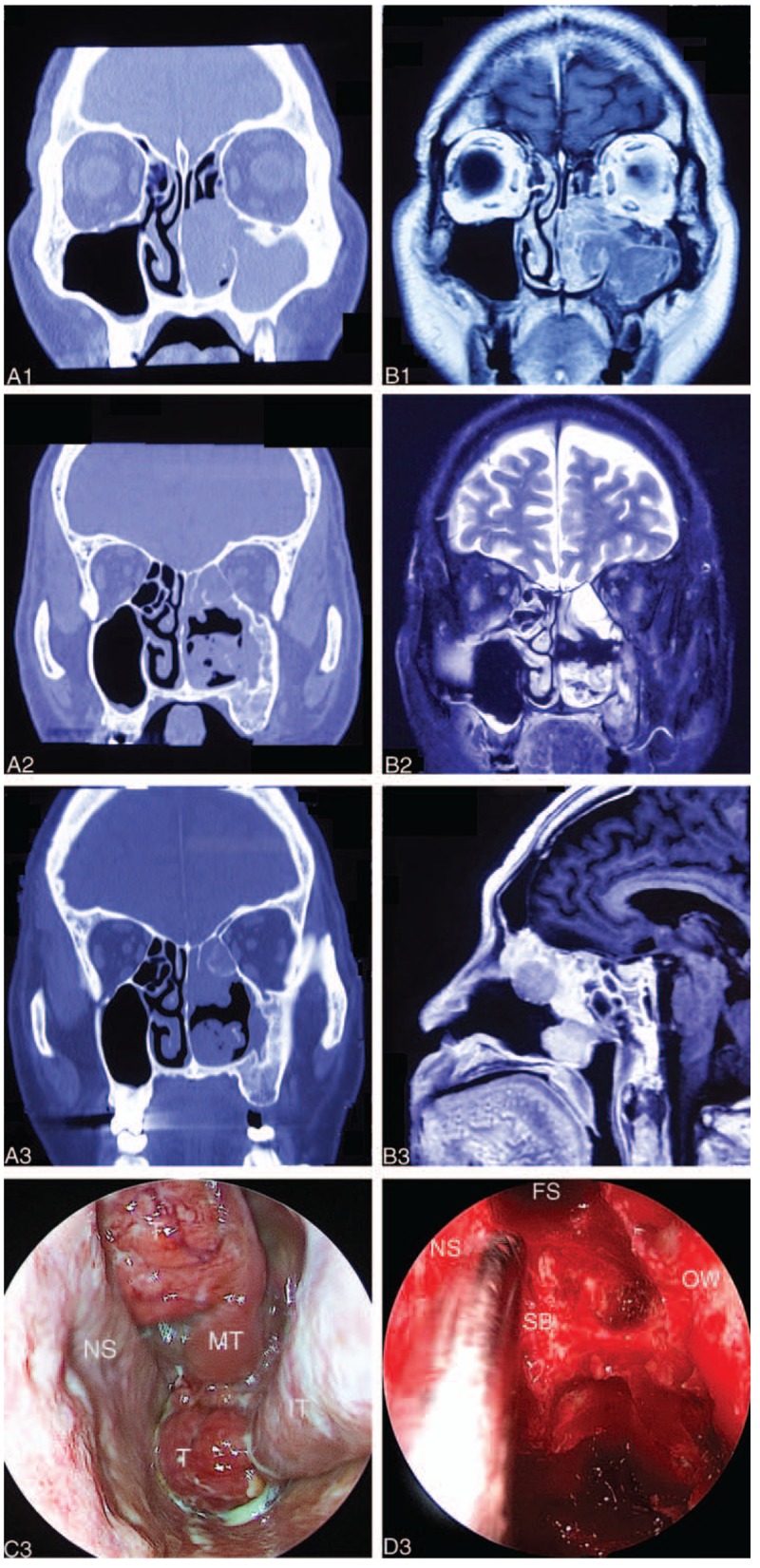
Imaging examination before the second (A1, B1), the third (A2, B2), and the fourth (A3, B3) surgery. The endoscopic view before and during the fourth surgery (C3, D3). (A1) Computed tomography (CT) scan showing osteitis around the left suborbital nerve canal (black arrow). (B1) T1-weighted, contrast-enhanced, fat-suppressed coronal MRI showing a massive left maxillary sinus neoplasm (black arrow). Note the striated appearance of the tumor. (A2) CT showing inflammatory response of the left maxillary bone (black arrow). (B2) MRI showing occupying lesion in the left nasal cavity, with absence of bony erosion into the orbit or skull base (black arrow). (A3, B3) CT and magnetic resonance images were similar to the last presurgery images. High inflammatory response of the left maxillary bone and 2 parts of the tumor were observed. Also, there was no bony erosion into the orbit or skull base. (C3) An endoscopic view before the fourth surgery. A neoplasm was located in the left nasal cavity, with a rough surface, easy bleeding, and more pus secretions on the surface of the tumor. (D3) Endoscopic intraoperative views showing anterior skull base exposure. FS = frontal sinus, IT = inferior turbinate, MT = middle turbinate, NS = nasal septum, OW = orbital wall, SB = skull base, T = tumor.

At the age of 79 years (in 2015), the patient presented with similar symptoms at Beijing Tongren Hospital, Beijing. Physical examination revealed the presence of a polyploid mass partially filling the middle meatus with extension into the nasal cavity. Computed tomography (CT) and magnetic resonance imaging (MRI) examination of the paranasal sinus (Fig. 1) revealed unilateral maxillary sinus disease, left orbital suborbital nerve canal hyperostosis, and findings consistent with inverted papilloma. Endoscopic sinus surgery was performed under general anesthesia. Intraoperative frozen pathology revealed carcinoma arising in inverted papilloma. The hyperostosis of the suborbital nerve canal of the maxillary sinus was removed and the margins of resection ended up positive. However, postoperative permanent pathology demonstrated carcinoma arising in OSP. The patient was recommended to have radiotherapy for the primary site of the tumor, but he declined.

The patient presented at age 81 years (in 2017) to Anzhen Hospital in Beijing with similar obstructive symptoms. CT and MRI (Fig. 1) revealed inflammatory response of the left maxillary bone and a space occupying lesion of the left nasal cavity. A soft tissue density mass was found in the left nasal cavity and sinus, with absence of bony erosion into the orbit or skull base. Endoscopic sinus surgery with surgical navigation under general anesthesia was performed with complete removal of the tumor and negative margins by frozen section. Postoperative histopathology was high-grade nonintestinal adenocarcinoma. The patient still refused to undergo radiotherapy after surgery.

**Figure 2:**
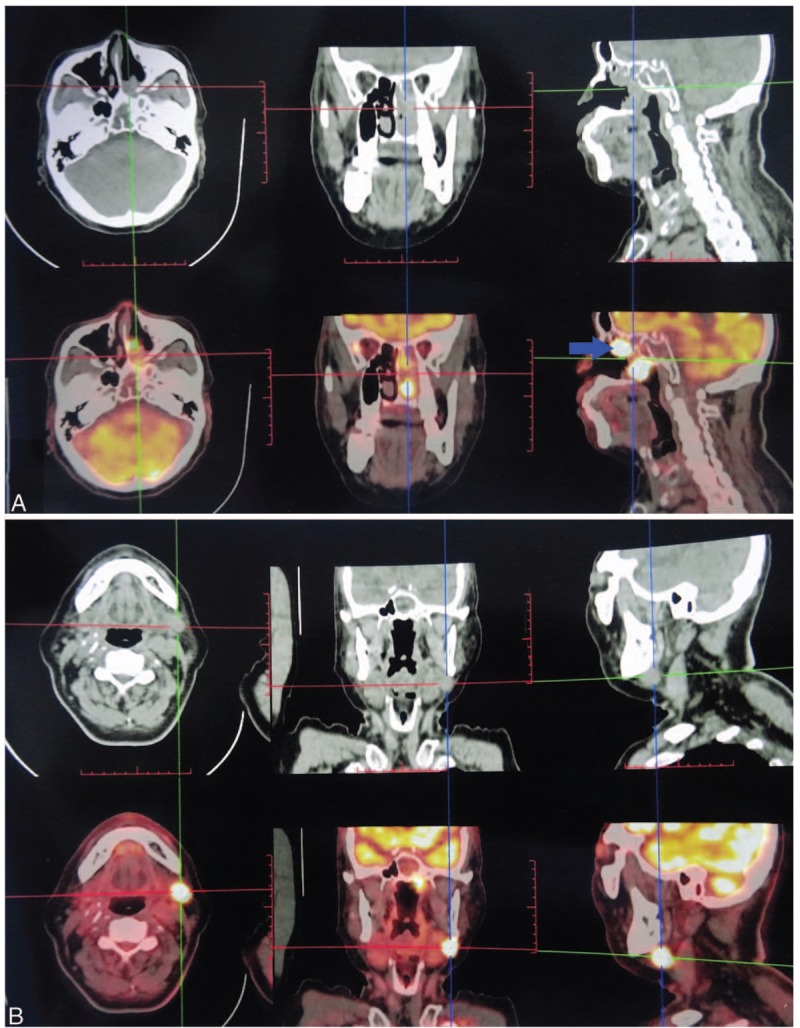
F-18 fluorodeoxyglucose-positron emission tomography/computed tomogramphy image before the fourth surgery. (A) The glucose metabolism of the left posterior nasal passages was increased (red arrow). The size of the mass was about 2.6 × 2.5 × 1.8 cm, and the density was not even. The CTavg was about 44 to 51 Hu and SUVmax 33 Hu. The radiation-uptake of the top of the left nasopharyngeal cavity was very thick; the size of the mass was about 2.5 × 2.4 × 1.7 cm, with CTavg about 45 to 53 Hu and SUVmax 45 Hu (blue arrow). (B) The enlarged lymph nodes in the left posterior mandible (black arrow) showed abnormal increase of glucose metabolism, about 2.2 × 2.0 × 1.8 cm and SUVmax 41 Hu. CTavg = computed tomography average value, SUVmax = maximum standard uptake values.

After 5 months, the patient returned and physical examination showed a neoplasm in the left nasal cavity, with a rough surface, easy bleeding, and purulent secretions on the surface of the tumor. In addition, there were palpable enlarged, hard, immbolile submandibular lymph nodes on that side (2.2 × 2.0 × 1.8 cm). CT, MRI, enhanced MRI (Fig. 1), and positron emission tomography-computed tomogramphy (Fig. 2) were performed and revealed 2 masses in the nasal cavity. One was located at the bottom of the nose and extended along the lateral wall of the nasal cavity to cover the pharynx and the eustachian tube, violating the root of the inner part of the pterygoid muscle. The other was located in the top of the nasal cavity, occupying the ethmoid sinus and the anterior skull base, but not invading the orbit and skull base bone. Metastatic swollen lymph nodes were seen under the left mandible. Endoscopic surgery was performed again with surgical navigation, resecting the tumor completely with negative frozen margins. We also performed a selective left neck dissection. Postoperative pathology of the primary tumor was low-differentiated adenocarcinoma and we found metastatic adenocarcinoma with low-differentiation in the cervical lymph nodes. The patient disagreed to receive adjuvant radiotherapy and targeted drug therapy for *KRAS* gene.

During 6 months’ follow-up after the last operation, the patient developed an in situ tumor recurrence 1 month after the fourth operation and refused to undergo surgery again.

### 2.3 Tissue preparation

Pathological sections were collected from all 3 hospitals after 3 operations, including the primary site and the metastatic lymph nodes. All diagnoses were confirmed centrally by an experienced head and neck pathologist (JBM).

### 2.4 Immunohistochemistry

Immunohistochemical studies were performed on 4-μm thick paraffin sections according to routine protocols using the EnVision method. The antibodies used for analyses are listed in Table 2. All antibodies were monoclonal; heat-induced epitope retrieval was performed by heating in Tris-EDTA buffer, pH 9.0. Immunostaining was reviewed by a single pathologist. Results were considered positive when >30% of tumor cells were stained.

**Table 2:**
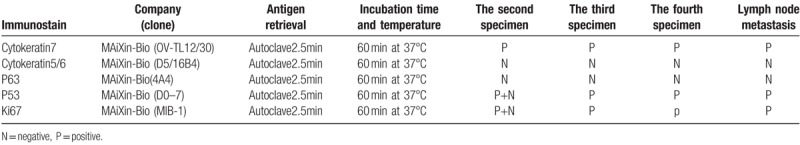
Immunohistochemistry results for tumors of the primary site and lymph node metastasis.

### 2.5 HPV assay

The BioPerfectus Multiplex Real Time (BMRT) HPV assay (Bioperfectus Limited Corp., TaiZhou, China) was developed to detect 13 high-risk (HR) HPV types, 5 medium-risk (MR) types, and 3 low-risk (LR) HPV types, as well as the viral loads simultaneously. PCR primers and corresponding TaqMan probes were designed to detect each of the 21 most prevalent HPV types, including 15 HR-HPV genotypes of HPV-16, -18, -31, -33, -35, -39, -45, -51, -52, -56, -58, -59 and -68; 5 MR-HPV genotypes of HPV-26, -53, -66, -73, and -82; and 3 LR-HPV genotypes of HPV-6, -11, and -81. The PCR reactions for the 21 genotypes were divided into 7 groups and the eighth group represented the control group. The 8 reactions were performed simultaneously. PCR amplification was conducted in a total reaction volume of 20 μL, which comprised 2 μL DNA samples, 10 μL Platinum Quantitative PCR SuperMix-UDG, 10 pmol of each primer, and 1 to 5 pmol of each probe (FAM, VIC, and ROX dye). Perfectus Software v1.0 was used for genotyping and quantitative analysis of HPV nucleic acid (Bioperfectus Limited Corp., China).

### 2.6 DNA sequencing

Targeted next-generation sequencing was performed. In brief, sequencing libraries were generated from 10 ng of extracted DNA using the AmoyDx FFPE DNA Kit (Stratagene Mx3000p). Conventional bidirectional Sanger sequencing of KRAS exons 2, 3, 4 and EGFR exons 18, 19, 20, and 21 were performed using nested sequencing primers (ADx-ARMS-EGFR, KRAS). The detection method can detect a mutation of up to 1% in the sample.

## 3 Result

### 3.1 Pathologic findings and immunohistochemical features

**Figure 3:**
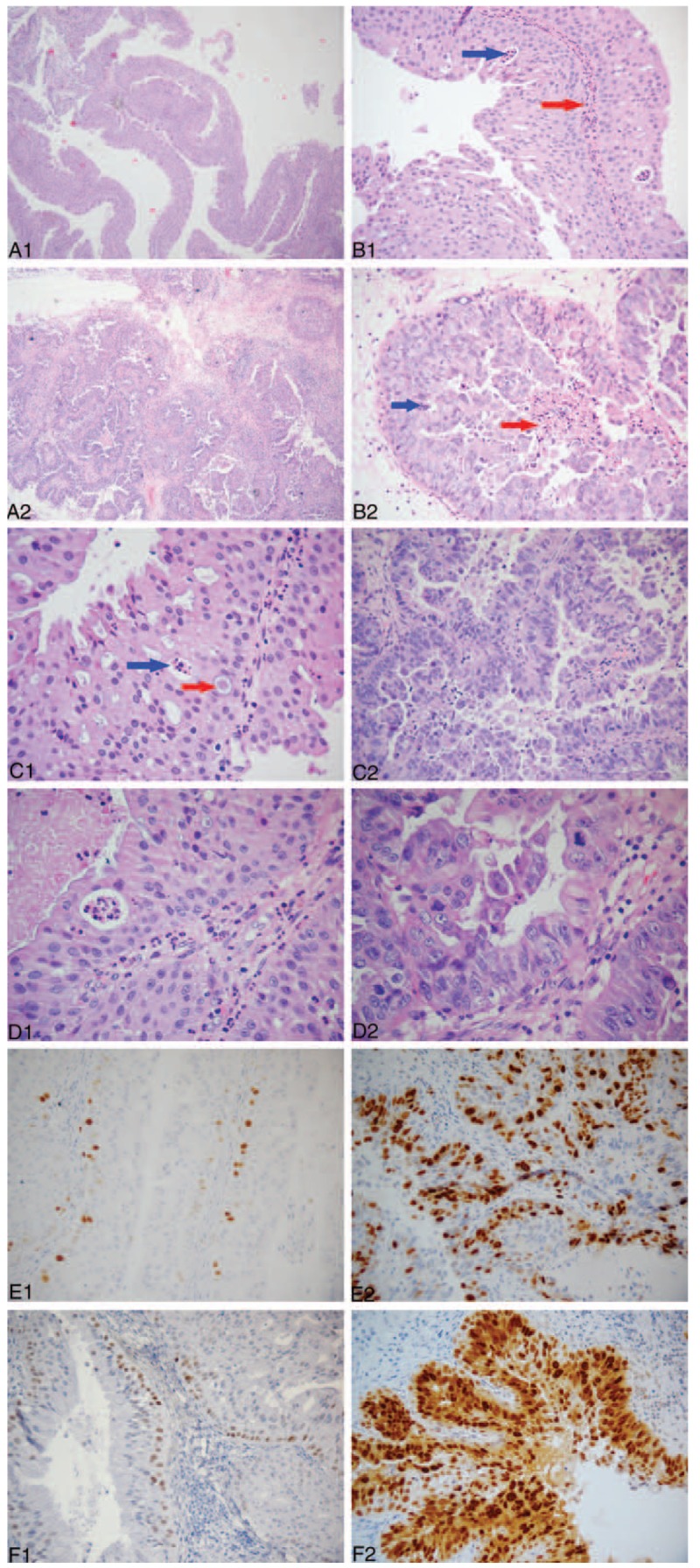
Pathological characteristics and immunohistochemical findings in the tumor in the second surgery. (A1) Low-power view showing the predominantly papillary surface pattern of the tumor. The cells are acidic. (B1) Medium-power view showing the microabscess structure of neutrophils with neatly arranged cells and no atypia. The blue arrow shows a microabscess and the red arrow indicates fibrous vascular axis. (C1) Scattered mucous cells and small cavities were visible under high-power view, and the cells were not heterotypic. The blue arrow shows a microabscess and the red arrow indicates a mucous cell. (D1) The structure is clearer under higher magnification. (E1) Ki67 staining of the tumor specimen. The proliferation index is low and only the basal layer cells are positive, indicating that the growth of the tumor is slow. (F1) p53 staining of the tumor specimen. Only the basal layer cells were positive, but the surface cells were negative, indicating that the cells were benign. Additional pathological characteristics and immunohistochemical findings in the tumor in the second surgery. (A2) Low-power view showing the predominantly papillary surface pattern of the tumor. Part of the tumor shows formation of small clusters, with crowded cells and the layered structure disappears. Visible infiltration of gland tubular structure in the stroma. (B2) Medium-power view showing glandular fusion, obvious cell atypia and visible nucleolus. The blue arrow shows a pathological mitosis-like nucleus, and the red arrow indicates necrosis/nuclear fragmentation. (C2) High-power view showing that the cells were crowded, disordered and heterotypic. (D2) Higher magnification of the mitotic-like nucleus. (E2) Ki67 staining indicated a high proliferation index. (F2) p53 staining of the tumor cells was positive, suggesting cell malignancy.

At the first resection, there was a pathological diagnosis of nasal polyps; unfortunately, these slides could not be found for analyses. At the second resection (Fig. 3), part of the tumor tissue supported the diagnosis of OSP, displaying a papillary structure and oncocytic appearance under low magnification. Observation under medium magnification showed that the microabscess structure was constructed by neutrophils and the cells were arranged orderly, and there were no heteromorphic features. Scattered mucous-secreting cells and small cavities were visible under high magnification, and the cells were not heterotypic. The tumor consisted of a bland, papillary, and inverted neoplasm. The cells had abundant, eosinophilic cytoplasm. Mitotic activity, however, was low in this area. The proliferation index was low and the basal layer cells were positive, indicating that the growth of the tumor was slow. There was no significant apoptosis and there was no necrosis. Immunohistochemical staining for p53 was positive only in the basal layer, indicating that the cells were benign.

However, another part of the tumor tissue supported the diagnosis of adenocarcinoma arising in OSP. At low magnification, the papillary structure was displayed in a small cluster. The cells were crowded and the layered structure was absent. Obvious cell atypia and the nucleoli were clearly visible under medium magnification. Under high magnification, the cells were crowded and disorderly, and the cells were heteromorphic. In addition to the papillary structure, infiltration of the gland tubular structure was visible in the interstitium. There was significant apoptosis and necrosis in this area. Mitotic activity, however, was high in this resected specimen. Immunohistochemistry for Ki67 showed a progression of staining across the 2 areas and an increase in mitotic activity. Immunohistochemical staining for p53 was positive. The overall pathological analysis of the resected tumor tissue showed that the lesions conformed to OSP. The formation and development of adenocarcinoma could be seen in some areas at the same time.

**Figure 4:**
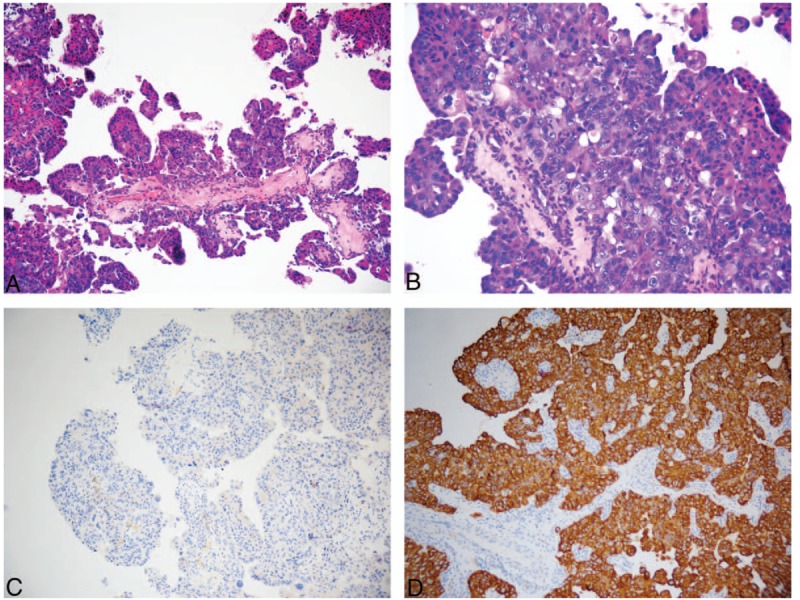
Pathological characteristics and immunohistochemical findings in the tumor in the third surgery. (A) Low-power view showing the predominantly papillary surface pattern of the tumor and cell congestion. (B) Medium-power view showing an increase in number of cell layers, and the cells were crowded and heterotypic. (C) Negative staining for the CK5/6 squamous cell carcinoma markers. (D) Positive staining for the CK7 adenocarcinoma markers.

At the third resection, observation under low magnification showed the structure of the nipple and crowding of the cells. At medium magnification, the cell levels were increased; the cells were crowded and the cells were obviously heterotypic (Fig. 4). Immunohistochemistry for CK5/6 and p63 was negative, but CK7 staining was positive. Thus, the tumor was adenocarcinoma, not squamous cell carcinoma.

**Figure 5:**
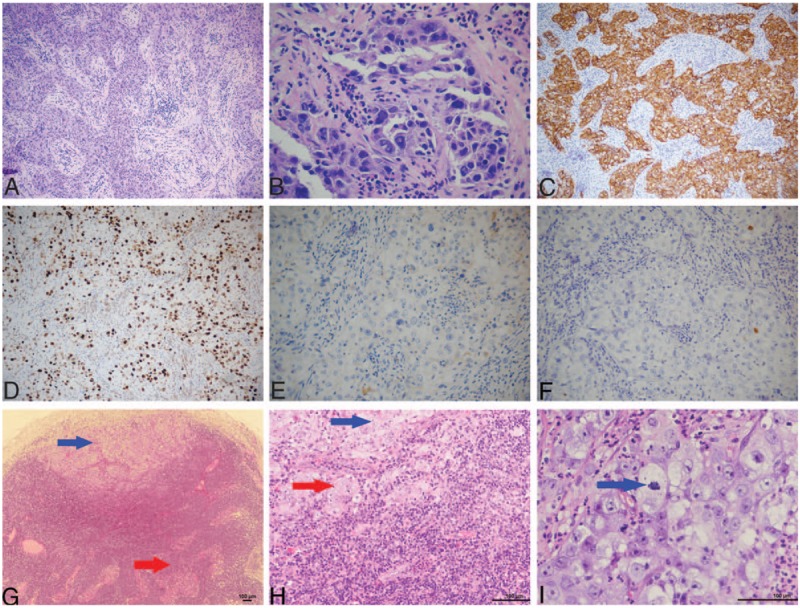
Pathological characteristics and immunohistochemical findings in the tumor in the fourth surgery. Metastasis tumor of cervical lymph nodes after the fourth operation. (A) Low-power view showing, in some regions, a solid arrangement of tumor cells and a low differentiated carcinoma structure. (B) High-power view showing obvious cell atypia. (C) Positive CK7 adenocarcinoma marker staining. (D) Ki67 staining indicated a high proliferation index, indicating actively proliferating cancer cells. (E) CK5/6 and (F) p63 stainings in the tumor showed negative staining for squamous cell carcinoma markers. (G) The blue arrow shows metastasis of the tumor in the marginal sinus of the lymph node. The red arrow indicates residual lymphoid tissue. (H) The blue arrow indicates a small number of tumor cells that are arranged in a gland tubular structure. The red arrow indicates that most of the tumor cells are flaky. (I) The nucleus is large, and it is vacuolated and polymorphous. The nucleoli are obvious, and mitotic image is visible. The blue arrow shows a mitotic image.

At the fourth resection (Fig. 5), low magnification images revealed interstitial infiltration of the gland ducts and the fibrous connective tissue around the ducts. In some regions, there was a solid arrangement of tumor cells and a low-differentiated carcinoma structure. There was obvious cell atypia under high magnification. Immunohistochemistry for CK5/6 and p63 was negative and staining for CK7 was positive. Immunohistochemistry for Ki67 showed that the proliferation index was high and the cancer cells were actively proliferating.

In analyzing the pathological changes, we found that the patient developed high-differentiated adenocarcinoma from OSP at first, and then moderately differentiated adenocarcinoma, and finally poorly differentiated or undifferentiated carcinoma. The entire process occurred over 3 years, and the recurrence interval was shorter over time, with an eventual appearance of lymph node metastasis.

### 3.2 HPV phenotypes

Both the initial resection specimen and the late course lymph node metastasis were negative for the 21 most prevalent HPV types.

### 3.3 DNA sequencing

Among KRAS exons 2, 3, and 4 and EGFR exons 18, 19, 20, and 21, only *KRAS* gene exon 2 G12C showed a mutation. No mutation was observed in the EGFR gene.

## 4 Discussion

Schneiderian papillomas are not common and OSPs are even more rare. IPs and OSPs are associated with a low risk for malignancy, of approximately 5% to 10%.^[4,8,11,12,15–18]^ This is well established and is reported to occur through the progression of squamous metaplasia to squamous dysplasia and then carcinoma.^[2,7]^ However, no definite case of adenocarcinoma with increasing malignant features arising from OSPs has been reported to date.

Here, we present a case of adenocarcinoma arising from OSP, according to pathological review. A papilloma is classified as OSP based on the presence of an epithelium composed of multiple layers of tall, columnar oncocytic epithelium with both exophytic and inverted growth patterns, up to 2 to 8 cells thick. The pathological features of OSP include cells with abundant, eosinophilic cytoplasm and intraepithelial microabscess that contains mucus and neutrophils.^[3,11]^ In the second relapse of the tumor in the present case, a typical feature of OSP was found in some areas by microscopy. In some areas, except for the papillary structure, there was an infiltration of an adenoid tube structure in the stroma, and pathological mitosis, necrosis, and nuclear fragmentation were observed. Combined with clinical and pathological features, this suggests that synchronous adenocarcinoma was arising from OSP. These were not 2 independent diseases. In addition, the degree of malignancy in the adenocarcinoma changed over recurrence and surgery.

The initial features described here were not typical for any defined Schneiderian papilloma and this case was misdiagnosed as a nasal polyp. This suggests that we should take multiple biopsies for intraoperative frozen pathological examination for unilateral polypoid lesions to prevent misdiagnosis. The relative rarity of OSP prevents comparison between OSP and IPs for local recurrence and malignancy coexistence.^[19]^ Here, we find that OSP is similar to IP by reviewing the clinical and radiological characteristics of this disease. For example, the lesions are located in unilateral sinus, with a pale pink appearance. Also, the hyperplasia of bone at the root of the tumor was detected by CT and via MRI. The second recurrence was diagnosed as oncocytic papilloma and concurrent with adenocarcinoma according to the final pathology. However, at that time, clinical and intraoperative frozen pathological results supported IP. The tumor recurred 4 times over 13 years, despite extensive surgical resection, including removal of the vertical plate of palatine bone, root of pterygoid muscle, and lymph node neck dissection. Throughout the patient's entire clinical course, the malignant features of the tumor were increasing, which manifested in the shorter time interval of recurrence, increased mitotic activity, and lymph node metastases.

In particular, regarding the pathological features of the third relapse, if no previous pathological analysis had been available, it would have been difficult to make an accurate diagnosis from only these results. As with all complex cases and recurrences, a diagnosis should consider previous pathology results; this is often a challenge, especially for this patient who was treated at 3 different centers.

The exact relationship between HPV and the pathogenesis of papillomas, along with malignant transformations, is not clear. The literature shows that low-risk and high-risk HPV can be found in a significant minority of IPs (usually high-risk HPV types) and the majority of EP (usually low-risk HPV types).^[5]^ However, low-risk and high-risk HPV has not been found in OSP.^[5]^ Both the initial resection specimen and the late course lymph node metastasis from this rare case were also HPV-negative.

Highly prevalent activating EGFR mutations were identified in the vast majority of IP cases and also are commonly present in ISP-associated sinonasal squamous cell carcinoma but not in ESP, OSP, or sinonasal squamous cell carcinoma without a known papilloma association.^[20]^ Some studies have indicated that KRAS mutations may be a disease-defining molecular feature of sinonasal oncocytic tumors.^[21]^ In this case report, KRAS mutations were found in the OSP-associated adenocarcinoma. The presence of KRAS mutations in this tumor indicates that EGFR-targeted therapy is likely to be ineffective.

## 5 Conclusions

This is the first case of adenocarcinoma of nasal cavity and paranasal sinuses with increasing malignant features arising from OSP in the literature to our knowledge. The tumor ultimately invaded deep soft tissues, with a 13-year period of progressive disease recurrence with lymph node metastases. This tumor appears to represent a unique type of sinonasal adenocarcinoma, which is associated with OSP. This patient illustrates the idea that progression from OSP to OSP-associated adenocarcinoma may take years, but once OSP has been transformed into adenocarcinoma, it shows increasing malignant features. This is also the first report of somatic KRAS mutation in OSP-associated adenocarcinoma. KRAS-targeted therapy may play a therapeutic role in the treatment of this rare disease.

## Acknowledgements

The authors express their sincere appreciation to the pathology staff from the Laboratory of Beijing Tong Ren Hospital and Anzhen Hospital for their excellent work on the immunostains for this case. The authors also thank Lin Xu, MD, for her design of BMRT HPV assay and for providing laboratory support for this testing. Jayant M. Pinto, M.D., F.A.C.S., Section of Otolaryngology-Head and Neck Surgery, The University of Chicago, provided useful input.

## Author contributions

LZ collected the clinical data and followed up with the patients, and wrote the manuscript. XZ, WY, and YW collected the pathological pictures. HL completed professional pathological analysis. DW revised the manuscript. YW designed the study and revised the manuscript. All authors have read and approved the final manuscript.

**Conceptualization:** Yongxiang Wei.

**Data curation:** Dong Chen, Wei Yu, Ying Wu, Honggang Liu, Yongxiang Wei.

**Formal analysis:** Xiping Li, Honggang Liu, Yongxiang Wei.

**Investigation:** Haili Sun, Qianwen Lv, Ping Zhang.

**Methodology:** Yongxiang Wei.

**Resources:** Chunhua Hu, Xiaodan Zheng.

**Validation:** Yongxiang Wei.

**Writing – original draft:** Lichuan Zhang, Chunhua Hu.

**Writing – review & editing:** Dawei Wu, Honggang Liu, Yongxiang Wei.

## References

[1] Lampertico P Russell WO Maccomb WS Squamous papilloma of upper respiratory epithelium. Arch Pathol 1963;75:293–302. 13928338

[2] Thompson L World Health Organization classification of tumours: pathology and genetics of head and neck tumours. Ear Nose Throat J 2006;85:74. 16579185

[3] Barnes L Schneiderian papillomas and nonsalivary glandular neoplasms of the head and neck. Mod Pathol 2002;15:279–97. 11904343 10.1038/modpathol.3880524

[4] Hyams VJ Papillomas of the nasal cavity and paranasal sinuses. A clinicopathological study of 315 cases. Ann Otol Rhinol Laryngol 1971;80:192–206. 4323842 10.1177/000348947108000205

[5] Lawson W Schlecht NF Brandwein-Gensler M The role of the human papillomavirus in the pathogenesis of Schneiderian inverted papillomas: an analytic overview of the evidence. Head Neck Pathol 2008;2:49–59. 20614323 10.1007/s12105-008-0048-3 PMC2807546

[6] Jenko K Kocjan B Zidar N In inverted papillomas HPV more likely represents incidental colonization than an etiological factor. Virchows Arch 2011;459:529–38. 21912908 10.1007/s00428-011-1139-1

[7] Nudell J Chiosea S Thompson LD Carcinoma ex-Schneiderian papilloma (malignant transformation): a clinicopathologic and immunophenotypic study of 20 cases combined with a comprehensive review of the literature. Head Neck Pathol 2014;8:269–86. 24519376 10.1007/s12105-014-0527-7 PMC4126921

[8] Ward BE Fechner RE Mills SE Carcinoma arising in oncocytic Schneiderian papilloma. Am J Surg Pathol 1990;14:364–9. 2321700 10.1097/00000478-199004000-00008

[9] Nachtigal D Yoskovitch A Frenkiel S Unique characteristics of malignant schneiderian papilloma. Otolaryngol Head Neck Surg 1999;121:766–71. 10580235 10.1053/hn.1999.v121.a98734

[10] Kapadia SB Barnes L Pelzman K Carcinoma ex oncocytic Schneiderian (cylindrical cell) papilloma. Am J Otolaryngol 1993;14:332–8. 8238761 10.1016/0196-0709(93)90091-k

[11] Barnes L Bedetti C Oncocytic Schneiderian papilloma: a reappraisal of cylindrical cell papilloma of the sinonasal tract. Hum Pathol 1984;15:344–51. 6370825 10.1016/s0046-8177(84)80033-7

[12] Yang YJ Abraham JL Undifferentiated carcinoma arising in oncocytic Schneiderian (cylindrical cell) papilloma. J Oral Maxillofac Surg 1997;55:289–94. 9054920 10.1016/s0278-2391(97)90545-0

[13] Maitra A Baskin LB Lee EL Malignancies arising in oncocytic schneiderian papillomas: a report of 2 cases and review of the literature. Arch Pathol Lab Med 2001;125:1365–7. 11570918 10.5858/2001-125-1365-MAIOSP

[14] Lewis JS Chernock RD Haynes W Low-grade papillary schneiderian carcinoma, a unique and deceptively bland malignant neoplasm: report of a case. Am J Surg Pathol 2015;39:714–21. 25634744 10.1097/PAS.0000000000000390

[15] Kaufman MR Brandwein MS Lawson W Sinonasal papillomas: clinicopathologic review of 40 patients with inverted and oncocytic schneiderian papillomas. Laryngoscope 2002;112 (8 pt 1):1372–7. 12172247 10.1097/00005537-200208000-00009

[16] Kristensen S Vorre P Elbrønd O Nasal Schneiderian papillomas: a study of 83 cases. Clin Otolaryngol Allied Sci 1985;10:125–34. 4028474 10.1111/j.1365-2273.1985.tb01181.x

[17] Phillips PP Gustafson RO Facer GW The clinical behavior of inverting papilloma of the nose and paranasal sinuses: report of 112 cases and review of the literature. Laryngoscope 1990;100:463–9. 2184302 10.1288/00005537-199005000-00004

[18] Vrabec DP The inverted Schneiderian papilloma: a 25-year study. Laryngoscope 1994;104 (5 pt 1):582–605. 8189990 10.1002/lary.5541040513

[19] Michaels L Young M Histogenesis of papillomas of the nose and paranasal sinuses. Arch Pathol Lab Med 1995;119:821–6. 7668940

[20] Udager AM Rolland DCM McHugh JB High-frequency targetable EGFR mutations in sinonasal squamous cell carcinomas arising from inverted sinonasal papilloma. Cancer Res 2015;75:2600–6. 25931286 10.1158/0008-5472.CAN-15-0340 PMC4508878

[21] Udager AM McHugh JB Betz BL Activating KRAS mutations are characteristic of oncocytic sinonasal papilloma and associated sinonasal squamous cell carcinoma. J Pathol 2016;239:394–8. 27234382 10.1002/path.4750

